# Prioritizing attributes of approaches to analyzing patient-centered outcomes that are truncated due to death in critical care clinical trials: a Delphi study

**DOI:** 10.1186/s13063-024-08673-x

**Published:** 2025-01-10

**Authors:** Melanie Bahti, Brennan C. Kahan, Fan Li, Michael O. Harhay, Catherine L. Auriemma

**Affiliations:** 1 Palliative and Advanced Illness Research (PAIR) Center, Perelman School of Medicine, University of Pennsylvania, Philadelphia, PA USA; 2 MRC Clinical Trials Unit at UCL, Institute of Clinical Trials and Methodology, University College London, London, UK; 3 Department of Biostatistics, Yale University School of Public Health, New Haven, CT USA; 4 Center for Methods in Implementation and Prevention Science, Yale University School of Public Health, New Haven, CT USA; 5 Department of Biostatistics, Epidemiology, and Informatics, Perelman School of Medicine, University of Pennsylvania, Philadelphia, PA USA; 6 Division of Pulmonary and Critical Care Medicine, Perelman School of Medicine, University of Pennsylvania, Philadelphia, PA USA; 7 Leonard Davis Institute of Health Economics, University of Pennsylvania, Philadelphia, PA USA

**Keywords:** Patient-centered outcomes, Truncation due to death, Delphi study, Consensus, Composite outcomes, Statistical approaches, Estimands

## Abstract

**Background:**

A key challenge for many critical care clinical trials is that some patients will die before their outcome is fully measured. This is referred to as “truncation due to death” and must be accounted for in both the treatment effect definition (i.e. the estimand), as well as the statistical analysis approach. It is unknown which analytic approaches to this challenge are most relevant to stakeholders.

**Methods:**

Using a modified Delphi process, we sought to identify critical attributes of analytic methods used to account for truncation due to death in critical care clinical trials. The Delphi panel included stakeholders with diverse professional or personal experience in critical care-focused clinical trials. The research team generated an initial list of attributes and associated definitions. The attribute list and definitions were refined through two Delphi rounds. Panelists ranked and scored attributes and provided open-ended rationales for responses. A consensus threshold was set as ≥ 70% of respondents rating an attribute as “Critical” (i.e., score ≥ 7 on a 9-point Likert scale) and ≤ 15% of respondents rating the measure as “Not Important” (i.e., a score of ≤ 3).

**Results:**

Thirty-one (91%) of 34 invited individuals participated in one or both rounds. The response rate was 82% in Round 1 and 85% in Round 2. Participants included eight (26%) personal experience experts and 26 (84%) professional experience experts. After two Delphi rounds, four attributes met the criteria for consensus: accuracy (the approach will identify effects if they exist, but will not if they do not), interpretability (the approach enables a straightforward interpretation of the intervention’s effect), clinical relevance (the approach can directly inform patient care), and patient-centeredness (the approach is relevant to patients and/or their families). Attributes that did not meet the consensus threshold included sensitivity, comparability, familiarity, mechanistic plausibility, and statistical simplicity.

**Conclusions:**

We found that methods used to account for truncation due to death in the treatment effect definition and statistical approach in critical care trials should meet at least four defined criteria: accuracy, interpretability, clinical relevance, and patient-centeredness. Future work is needed to derive objective criteria to quantify how well existing estimands and analytic approaches encompass these attributes.

**Supplementary Information:**

The online version contains supplementary material available at 10.1186/s13063-024-08673-x.

## Background

There is widespread agreement around the importance of evaluating non-mortality outcomes that matter to patients and families, such as quality-of-life, functional status, or hospital length-of-stay, in trials for patients with critical illnesses [1–4]. However, in any trial with patients at high risk for mortality, some participants may die before these outcomes are measured. For example, for patients who die before the conclusion of a study, we cannot measure their end-of-study quality of life; similarly, for patients who die before hospital discharge, we cannot measure their time to discharge [5–10]. This is referred to as “truncation due to death”, and must be accounted for in both the treatment effect definition (i.e. the estimand [11–13]) as well as the statistical analysis approach, as failure to do so can lead to results that are biased, ambiguous, or misleading [5, 6, 10, 14, 15].

There exist various approaches to account for truncation due to death. For example, investigators could define a composite outcome that incorporates death, use a statistical model to estimate the treatment effect in those patients who would survive under either treatment, or estimate the effect had all patients survived [11, 12, 16]. These various approaches may have different advantages and disadvantages in terms of interpretability, clinical relevance, and ease of statistical estimation [11]. However, it is not clear which approaches are most relevant or useful to key stakeholders who make or are impacted by clinical decisions informed by trial results [17–19]. In circumstances in which there is an absence of agreement on a potentially controversial subject, formal consensus methods provide tools for defining levels of agreement among experts [20]. Such approaches have been applied to a wide range of topics and used to generate clinical and research guidance [21–25].

A key step before generating specific guidance for researchers on the optimal approach to account for truncation due to death in treatment effect definitions or statistical methods is to first understand which attributes of an approach are prioritized by stakeholders. To aid in the development of such guidance, we sought to reach expert consensus on critical attributes of approaches used to handle truncation due to death in intensive care clinical trials among international stakeholders.

## Methods

We conducted a two-round modified Delphi consensus process [20, 26, 27]. The modified Delphi process is an iterative approach that allowed stakeholders to (1) rate attributes of approaches for handling truncation due to death in critical care clinical trials; (2) suggest additional attributes to rate; (3) explain their ratings and review the explanations of other participants; and (4) revise responses in Round 2 by taking into account information learned in the prior round [27–29].

### Participant recruitment

We convened a panel of experts with professional or personal experience in critical care clinical trials. Professional experience experts included statisticians, clinicians, and clinical trialists. Professional experience experts were identified through the authorship team’s professional networks and leadership of critical care professional societies (American Thoracic Society, Society of Critical Care Medicine). Personal experience experts included critical illness survivors or family members who have served as patient advocates and representatives in clinical trial design or on patient-caregiver advisory committees for research grants. Personal experience experts were identified through the leadership of national critical illness and patient and family support groups (ARDS Foundation, ARDS Alliance). The target panel size was at least 18 individuals as a panel size of 18 experts has shown excellent ability to reach consensus [30, 31] and can help ensure increased engagement by participants as well as higher response rates over cycles. Prospective panelists were invited to participate directly by the authors via email and were informed that completing study surveys indicated informed consent to participate. We anticipated that approximately 60% of invited individuals would elect to participate in the Delphi panel.

### Survey design and administration

The overarching objective of this Delphi study was to identify critical attributes of approaches used to account for truncation due to death in critical care clinical trials. An initial list of seven attributes and associated definitions (Table 1) was generated by the authors through a review of key literature on methods to handle truncation by death both in critical care trials and beyond [5, 13, 32–35] as well as individual expert interviews with anticipated members of the Delphi panel prior to the initiation of the Delphi survey rounds [29, 36]. To engage participants from a wide range of educational and professional backgrounds, we worked within our multi-disciplinary research team to develop a video that described the objective of the study and relevant concepts using plain language and accompanying graphics (Supplement) [37, 38]. Participants were asked to view the video prior to initiating the Round 1 survey.

**Table 1:** Attribute definitions and participant responses

**Attribute**	**Round 1 Definition**	**Round 2 Definition**	**Participants Ranking Attribute as Most Important**^c^ n (%)	**Participants Ranking Attribute as Least Important**^**c**^ n (%)	**Points Allocated**^**c**^ Median (IQR)
Accuracy	The approach is statistically able to identify an effect if one exists, and won’t suggest an effect if one does not exist	*No changes*	18 (62%)	0 (0%)	20 (15, 25)
Clinical Relevance	The approach is clinically informative. That is, the result can directly inform clinical care	The approach provides an estimate of the effect of an intervention that could inform clinical care	6 (21%)	1 (3%)	15 (10, 17)
Patient-Centeredness	The approach has been shown to be relevant and/or important to patients and/or families	The approach provides an estimate of the effect of an intervention that is relevant and/or important to patients and/or families	2 (7%)	2 (7%)	15 (9, 19)
Interpretability	The approach enables a straightforward, simple, or clear interpretation of the results	The approach enables a straightforward, simple, or clear interpretation of the effect of an intervention	1 (3%)	1 (3%)	12 (5, 15)
Sensitivity	The approach has high statistical power to capture small effects and thus optimize sample size	The approach can capture small effects and thus optimize sample size	0 (0%)	5 (17%)	10 (5, 15)
Comparability^a^	The approach produces results that can be compared across different trials and studies	*NA*	*NA*	*NA*	*NA*
Familiarity^a^	The approach has been used in prior research	*NA*	*NA*	*NA*	*NA*
Mechanistic Plausibility^b^	*NA*	The approach enables a reasonable causal interpretation regarding the effect of an intervention	2 (7%)	8 (28%)	10 (5, 12)
Practicality^b^	*NA*	The approach places little to no additional burden on trial participants and investigators such that the data required (e.g., additional baseline or follow-up information) can be obtained with minimal intrusiveness and missingness	0 (0%)	1 (3%)	10 (5, 13)
Statistical Simplicity^b^	*NA*	The approach relies on a limited number of assumptions to estimate the effect of an intervention	0 (0%)	11 (38%)	10 (5, 12)

*Definition of abbreviations*: *NA* not assessed, *IQR* interquartile range

^a^Attribute removed for Round 2

^b^Attribute added for Round 2

^c^Task reported from Round 2

Anonymity of individual panelist’s responses was maintained throughout the Delphi process. The REDCap online survey platform was used to collect demographic information as well as to administer the Delphi surveys [39, 40]. Participants received up to 3 reminder emails to complete each survey round. The Delphi study was conducted between September 26, 2023, and November 18, 2023. This study was approved by the University of Pennsylvania IRB (protocol # 854392). The protocol was not registered prospectively. This study is reported in accordance with guidelines for consensus-based research (Supplementary Material) [41].

#### Round 1

In Round 1, participants reviewed the initial list of attributes and definitions. They were invited to suggest changes to the original definitions, propose up to three additional attributes for inclusion, and provide definitions for any newly proposed attributes. Participants were then asked to score the importance of the seven original attributes and any new attributes they proposed. Participants were also asked to compare the importance of the attributes relative to one another through a point allocation task. In this question, participants were instructed to distribute 100 points across the attributes to reflect their relative importance. Any individual attribute could receive 0–100 points, with the total across attributes required to sum to 100. Participants were asked to justify their quantitative responses to the survey in open-ended form.

#### Round 2

Following the completion of Round 1, participant responses were distributed to panelists in an executive summary that compiled anonymized responses from the prior survey (Supplement). Quantitative responses were presented graphically. Selected quotations from panel member comments were also included. Quotations were selected to ensure representation of comments across the range of scores for each attribute and to communicate any substantive concerns or reasoning raised by participants. We omitted comments that essentially reiterated a panelist’s score without including the reasoning for that score (e.g. “very important!”). Participants were asked to review the summary prior to completing the Round 2 survey.

The Round 2 survey was revised to exclude attributes that garnered minimal support in Round 1 and to add three new attributes that had been proposed by multiple participants. Original attribute definitions were also revised based on participant feedback. Round 2 repeated the attribute scoring and point allocation tasks, and additionally included an opportunity to rank attributes from least to most important. Again, participants were asked to justify their quantitative responses to the survey in open-ended form.

### Statistical reporting and analysis

Response rates were defined as the proportion of invited panel members who completed each survey. Quantitative responses were summarized using descriptive statistics. Questions regarding attribute importance were scored on a 9-point Likert scale ranging from 1 (“Not Important”) to 9 (“Critical”). Attributes were formally assessed for consensus following Round 2. Consensus threshold was set a priori as ≥ 70% of respondents rating the attribute as “Critical” (i.e., score ≥ 7) and ≤ 15% of respondents rating the attribute as “Not Important” (i.e., score of ≤ 3). Similar consensus definitions have been used in prior studies to ensure that an item will not achieve consensus if a subset of stakeholders commonly rates it as “Not Important” [21–23].

## Results

Thirty-four individuals were invited to participate in the Delphi. Thirty-one (91%) individuals participated in one or both rounds. Participants included eight (26%) personal experience experts and 26 (84%) professional experience experts (Table 2). Three panelists (10%) had overlapping personal and professional experience. Fifteen participants (48%) primarily practiced and/or worked in the United States, with the remainder in Europe (10, 29%), Canada (4, 13%), and Australia (2, 6%). Thirteen participants (42%) were female. Professional experience experts’ clinical specialties included adult and pediatric critical care as well as medical and anesthesia critical care. Clinical roles included physician, physical therapist, and nursing.

**Table 2:** Participant characteristics

Characteristic	Participants (*n* = 31)
Gender, n (%)	
Female	13 (42%)
Male	17 (55%)
Missing/Prefer not to answer	1 (3%)
Race, n (%)	
Asian	1 (3%)
Black/African American	1 (3%)
White	27 (87%)
Other: Southeast Asian, Iranian/Persian	2 (6%)
Ethnicity, n (%)	
Hispanic (Latino/Latina)	1 (3%)
Non-Hispanic	30 (97%)
Country, n (%)	
Australia	2 (6%)
Canada	4 (13%)
Denmark	3 (10%)
Germany	2 (6%)
Ireland (Republic)	1 (3%)
United Kingdom	4 (13%)
United States	15 (48%)
Professional Experience (*n* = 26)	
Role, n (%)	
Clinical trialist	16 (62%)
Statistician	12 (46%)
Clinician	10 (38%)
Other: journal editor, regulator, researcher	3 (12%)
Years Experience (mean, SD)	17 (11)
Personal Experience (*n* = 8)	
Role, n (%)	
Critical or serious illness survivor	4 (50%)
Family member or caregiver	4 (50%)
Patient or family member advocate	4 (50%)

*Definition of abbreviations*: *SD* standard deviation

Values represent number and percentage unless otherwise specified. Three participants identified as having both professional and personal experience

### Round 1

Round 1 was completed by 28 individuals (82% response rate). In Round 1 of the Delphi, participants scored seven attributes of approaches to handle truncation due to death in critical care clinical trials: accuracy, sensitivity, interpretability, clinical relevance, patient-centeredness, comparability, and familiarity (Table 1).

**Fig. 1:**
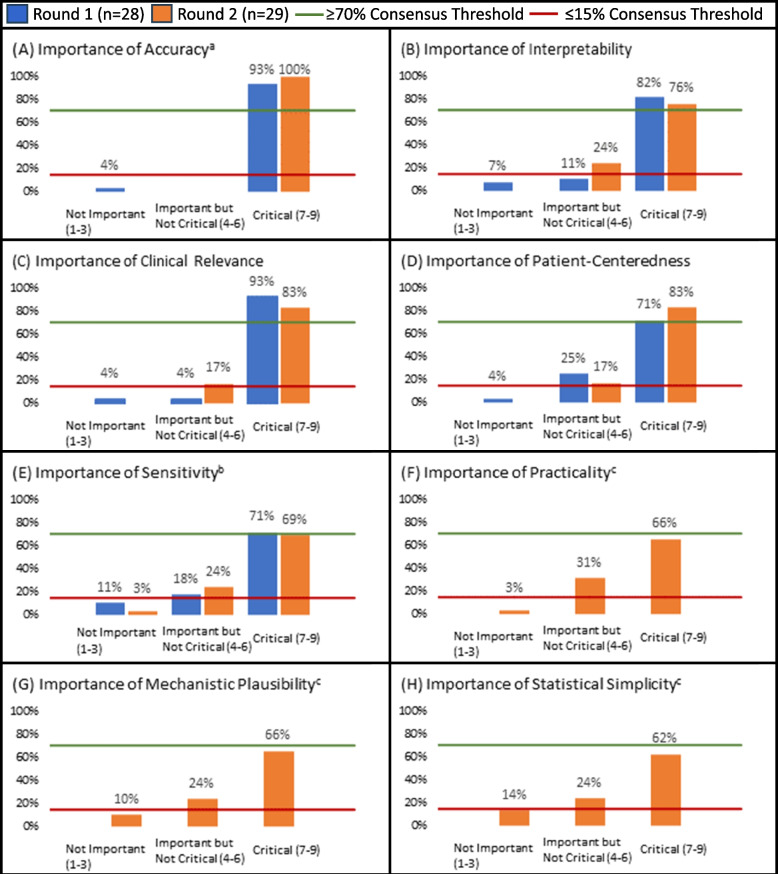
Importance of attributes for approaches used to analyze patient-centered outcome data missing due to death. **A** Importance of Accuracy. **B** Importance of Interpretability. **C** Importance of Clinical Relevance. **D** Importance of Patient-Centeredness. **E** Importance of Sensitivity. **F** Importance of Practicality. **G** Importance of Mechanistic Plausibility. **H** Importance of Statistical Simplicity. ^a^One participant responded as “Unable to score” for this attribute in Round 1. ^b^One participant responded as “Unable to score” for this attribute in Round 2. ^c^Attribute added for Round 2

Accuracy and clinical relevance received the greatest proportion of responses as “Critical” (*n* = 26, 93%), followed by interpretability (*n* = 23, 82%), sensitivity (*n* = 20, 71%), and patient-centeredness (*n* = 20, 71%). Comparability and familiarity each garnered lower support as “Critical” (*n* = 14, 50% and *n* = 7, 25%, respectively) and the highest proportions of participants rating as “Not Important” (*n* = 3, 11%, and *n* = 9, 32%, respectively) (Figure 1). Seventeen additional characteristics were proposed by panelists, reviewed by the study team, and consolidated into three new attributes: practicality, mechanistic plausibility, and statistical simplicity.

The number of points allocated to a single attribute in the point allocation task in Round 1 ranged from 0 to 50. Accuracy received the greatest median total of points (20 (inter-quartile range (IQR) 14.5, 25), followed by clinical relevance (17, IQR 13.5, 20), patient-centeredness (15, IQR 12, 22.5), interpretability (13, IQR 10, 15), sensitivity (10, IQR 9, 15), comparability (10, IQR 5, 10), and familiarity (5, IQR 0, 10).

Based on scoring and comments from Round 1, several attribute definitions were revised for clarity, and two attributes (comparability and familiarity) were dropped from inclusion in Round 2 due to low scores (Table 1).

### Round 2

Round 2 was completed by 29 individuals (85% response rate), 26 (90%) of whom had also completed Round 1. In Round 2 of the Delphi, participants scored the retained and new attributes from Round 1. Four attributes met the threshold for consensus (accuracy, interpretability, clinical relevance, and patient-centeredness), and were deemed critical qualities of an approach used to account for truncation due to death in critical care clinical trials (Figure 1). Four attributes did not meet the threshold for consensus: sensitivity, practicality, mechanistic plausibility, and statistical simplicity. One panelist did not provide a score in Round 2 for sensitivity, citing concerns with the attribute definition.

When asked to rank attributes from most to least important, accuracy (*n* = 18, 62%) was most commonly ranked as the most important attribute, followed by clinical relevance (*n* = 6, 21%), patient-centeredness (*n* = 2, 7%), and mechanistic plausibility (*n* = 2, 7%) (Table 1). No participants ranked statistical simplicity, sensitivity, or practicality as the most important attribute. Statistical simplicity (*n* = 11, 38%), mechanistic plausibility (*n* = 8, 28%), and sensitivity (*n* = 5, 17%) were most commonly ranked as the least important attribute.

The number of points allocated to a single attribute in the point allocation task in Round 2 ranged from 0 to 50. Accuracy received the greatest median total of points (20, IQR 15, 25), followed by clinical relevance (15, IQR 10, 17), patient-centeredness (15, IQR 9, 19), interpretability (12, IQR 10, 15), sensitivity (10, IQR 5, 15), statistical simplicity (10, IQR 5, 12), practicality (10, IQR 5, 13), and mechanistic plausibility (10, IQR 5, 12) (Table 1).

## Discussion

In this Delphi study, we established a diverse panel of stakeholders with expertise in critical care clinical trials to identify critical attributes of approaches used to handle truncation due to death. Through formal assessment of consensus, ranking, and point-allocation tasks, accuracy, interpretability, clinical relevance, and patient-centeredness consistently emerged as the top attributes. To our knowledge, this is the first empiric work to identify the key attributes for analyzing outcomes impacted by truncation due to death. Understanding which attributes stakeholders prioritize is a necessary step to ultimately evaluate perspectives on individual approaches, such as specific estimands or estimating approaches.

The attributes of sensitivity, practicality, mechanistic plausibility, and statistical simplicity all missed the predetermined threshold for consensus. While the definition of consensus used in this study is based on prior literature [21–23], a strict numerical threshold can still be considered somewhat arbitrary. Given the panel size in this study, scores from only one or two panelists made the difference between several attributes reaching consensus versus not. However, one of the strengths of this study is our incorporation of three distinct, but complementary, approaches to measuring prioritization of attributes: rating, ranking, and allocation tasks [42–44].

By integrating responses from Likert scales, rankings, and point-allocation tasks, our findings illustrate that while non-critical attributes are not *unimportant*, there is consistency that they are *less* important than accuracy, interpretability, clinical relevance, and patient-centeredness. The benefits of using multiple approaches to assess priorities also provides insight into minority opinions. For example, mechanistic plausibility, an attribute that was added to the list in Round 2 based on panelist feedback from Round 1, may seem to be controversial given its occurrence in the list of attributes ranked as both most *and* least important. Results from the rating and point allocation tasks, however, confirm this attribute to be of lower priority to most participants.

Accuracy, defined as “the approach is statistically able to identify an effect if one exists, and won't suggest an effect if one does not exist,” was rated as the most important attribute. In this study, we asked participants to rank attributes independent of specific estimands or modeling approaches, and “the effect” being estimated was not specified. Further efforts to investigate how well existing approaches to handling truncation due to death satisfy the prioritized attributes, including accuracy, should specify the particular effects being estimated with any chosen analytic approach.

This study has limitations. First, while our panel had broad international representation from North America and Western Europe, participation outside these regions and among minoritized racial and ethnic groups was modest. Alternative composition of panel members or including additional participants may have yielded different results. Second, while we provided plain language definitions of each attribute and incorporated participant feedback into the revised definitions distributed in Round 2, we did not formally assess participant comprehension, and definitions may remain imperfect. It is possible that some highly technical professional experience experts as compared to personal experience experts may have interpreted attributes differently. Future studies would benefit from ongoing refinement and validation of attribute definitions. Third, because the candidate list of attributes changed between rounds, we could not assess individual stability of responses.

## Conclusions

In summary, stakeholders with expertise in critical care clinical trials agree that methods to account for truncation due to death should be accurate, interpretable, clinically relevant, and patient-centered. This work underscores the importance of incorporating end-users’ perspectives in clinical trial design and suggests using these priorities to identify and evaluate optimal approaches for handling truncation due to death. Future work is needed to understand how well existing approaches, and in particular specific estimands or modeling strategies, encompass these critical attributes.

## Supplementary Information

Supplementary Material 1.

## Data Availability

The datasets used and/or analyzed during the current study are available from the corresponding author upon reasonable request.
