# Supplementary material for: Prioritizing attributes of approaches to analyzing patient-centered outcomes that are truncated due to death in critical care clinical trials: a Delphi study
Source: Trials. 2025 Jan 10;26:15. doi: 10.1186/s13063-024-08673-x (PMC11721323; doi:10.1186/s13063-024-08673-x)
Supplement: Supplementary file 1 — Supplementary Material 1. [file 13063_2024_8673_MOESM1_ESM.docx]

**Table of Contents**

1. Introductory video for Delphi participants
2. Round 1 Executive Summary^[[1]](#footnote-1)^
3. List of participants
4. Introductory Video - <https://youtu.be/gEZa1dRU3jc>
5. Round 1 Executive Summary

Delphi Round 1: Executive Summary

Thank you for participating in this PCORI-funded Delphi study, “Methods to analyze patient-centered outcomes missing due to death in cluster-randomized trials.” This executive summary presents the results from Round 1. We ask that you review these results to inform your participation in Round 2.

In reviewing Round 1 responses, numerical scores for each attribute were grouped into three categories: Not Important (scores of 1-3), Important but Not Critical (4-6), and Critical (7-9).

Based on your responses in Round 1, we have revised the Round 2 survey as follows:

- 5 attributes are retained, with some modifications to definitions:
  - Accuracy
  - Sensitivity
  - Interpretability
  - Clinical Relevance
  - Patient-Centeredness
- 3 attributes are added:
  - Practicality
  - Mechanistic Plausibility
  - Statistical Simplicity
- 2 attributes are dropped:
  - Comparability
  - Familiarity

In this report, we will show you a summary of the scores for each of the above attributes as well as representative quotations from participants’ free-text responses.

Background Refresher

In a clinical trial, scientists want to determine how an intervention (like a new treatment) may have impacted patients. In other words, they try to determine how well a new treatment worked.

In trials of very sick patients, some participants may die before the end of the study. The two broad approaches used to analyze outcomes when data is missing due to death are statistical models and composite outcomes.

These approaches are used to estimate the impact of the treatment, that is, the effect of the intervention. Scientists must choose an approach to making these estimates when they design and analyze clinical trials.

We want to develop a set of criteria to grade these approaches.

To do so, we want to:

1. Understand how you prioritize attributes, or characteristics, of possible approaches, and
2. Score a selected set of approaches along those attributes.

Round 2 focuses on finalizing Goal #1.

Accuracy

| **Round 1 Definition** | **Revised Definition** |
| --- | --- |
| The approach is statistically able to identify an effect if one exists, and won’t suggest an effect if one does not exist. | No changes. |


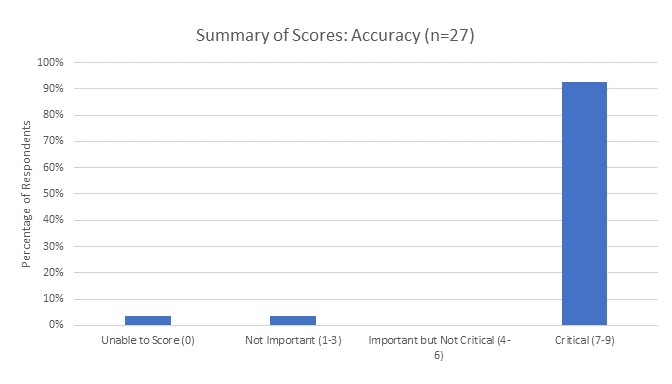


Representative comments:

| **Comment** | **Score** |
| --- | --- |
| Ability to detect true effects is crucial – otherwise the study will have limited value. | 9 |
| If you don’t know that there is a result, then everything else is moot. | 9 |
| Accuracy seems to have more to do with trial design, rather than the endpoint in itself. | 0 |

Sensitivity

| **Round 1 Definition** | **Revised Definition** |
| --- | --- |
| The approach has high statistical power to capture small effects and thus optimize sample size. | The approach can capture small effects and thus optimize sample size. |


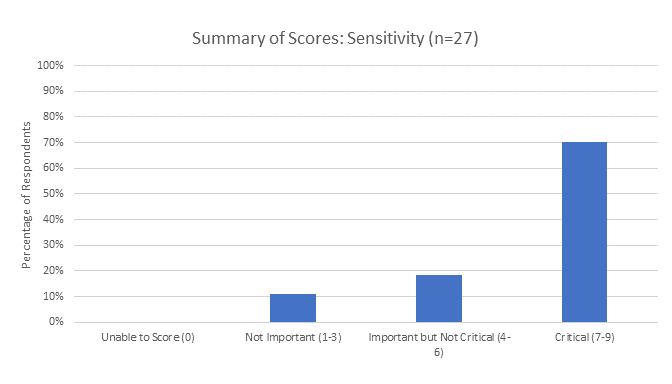


Representative comments:

| **Comment** | **Score Given** |
| --- | --- |
| Running trials with optimized sample sizes for efficiency is crucial, particularly in critical care settings or when dealing with rare diseases. | 9 |
| Then if you missed the effect, then everything else is moot. | 8 |
| In isolation, I think this point is important. However, it’s only important given other aspects, i.e., interpretability/clinical relevance/etc. I.e., there’s little value in using a measure/approach which makes it easy to identify an effect on an irrelevant measure. | 5 |
| Statistical power is important but if the effect measure is not relevant to decision makers, then statistical power becomes less relevant. For example, time-to-event analyses often have more power but reducing time to death for critical care studies has limited relevance to decision makers who want to save lives. | 5 |
| This is important, but is already encompassed by the definition of ‘accuracy’. | 3 |

Interpretability

| **Round 1 Definition** | **Revised Definition** |
| --- | --- |
| The approach enables a straightforward, simple, or clear interpretation of the results. | The approach enables a straightforward, simple, or clear interpretation of the effect of an intervention. |


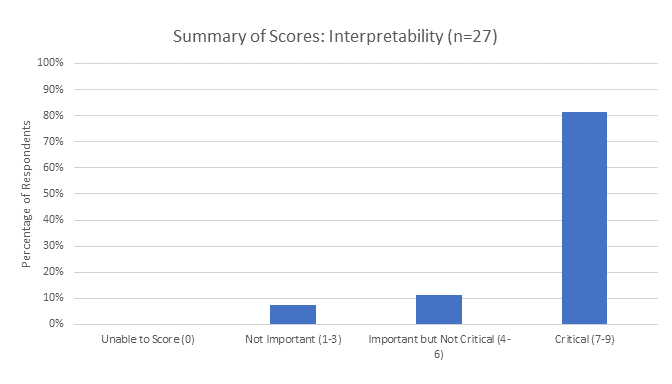


Representative comments:

| **Comment** | **Score** |
| --- | --- |
| I believe that without this attribute, the approach would be futile. The detected effect should be easily interpretable for researchers and the general public. | 9 |
| It is important that the trial yields results that are clear, understandable, and easy to apply for those utilizing these results. | 8 |
| Less relevant as an aim, as I feel many people interpret however they want, anyways, based on their own priors. | 7 |

Clinical Relevance

| **Round 1 Definition** | **Revised Definition** |
| --- | --- |
| The approach is clinically informative. That is, the result can directly inform clinical care. | The approach provides an estimate of the effect of an intervention that could inform clinical care. |


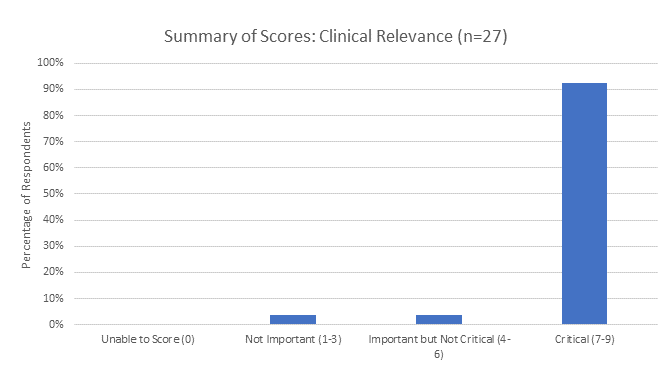


Representative comments:

| **Comment** | **Score** |
| --- | --- |
| Research must inform clinical care or it has very limited value. Therefore, it is crucial that an outcome supports changes in clinical care. | 9 |
| This is obviously important, but perhaps challenging to define exactly, e.g., whether the results can define patient care is to some extent a subjective decision made by those in charge of deciding patient care. | 8 |
| If there is a reliable finding, then does it matter? | 7 |

Patient-Centeredness

| **Round 1 Definition** | **Revised Definition** |
| --- | --- |
| The approach has been shown to be relevant and/or important to patients and/or families. | The approach provides an estimate of the effect of an intervention that is relevant and/or important to patients and/or families. |


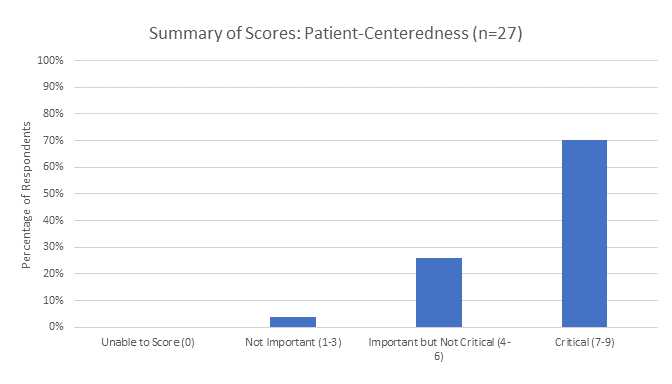


Representative comments:

| **Comment** | **Score Given** |
| --- | --- |
| If the research isn’t relevant and/or important to patients and families, then why do the study? It is not enough that clinicians and researchers are interested in certain outcomes and results if patients and families are not appropriately considered and their input is not included in all aspects of the research project. | 9 |
| Patients are becoming more empowered in decision making and thus ensuring the outcomes matter to patients is a crucial way of ensuring that research impacts care. The only reason this is less than 9 is that there may be some challenges in including the patient voice in critical care as many of the patients sadly die and are not able to provide information to researchers. | 8 |
| This is clearly important, though I think there’s a distinction between something that *is* important to patients, and something that has been *shown* to be important, e.g. something can be important to patients even if there hasn’t been formal research showing this. | 8 |
| Proof of concept/safety and reasonable NNT (in Phase 2/3 respectively) come first. Patient centeredness is a basket of effects that patients may weigh differently thus need context. Such outcomes are important secondary outcomes. | 5 |

Attributes Added Following Round 1:

A total of 17 new attributes were proposed during Round 1. We reviewed the definitions and explanations of these attributes and identified three common domains.

Practicality

| The approach places little to no additional burden on trial participants and investigators such that the data required (e.g., additional baseline or follow-up information) can be obtained with minimal intrusiveness and missingness. |
| --- |

Representative examples of proposed attributes contributing to our definition:

| **Proposed Attribute Name** | **Definition** |
| --- | --- |
| Burden | The burden upon patients, the healthcare system, or the research infrastructure required to ascertain the outcome. |
| Collectability | Collection of the outcome parameter is possible and feasible (without anticipating a lot of missing due to organizational issues). |
| Convenience to respondents | The approach enables "unintrusive" data collection, i.e., without disrupting the respondents' daily activities. |
| Ease of measurement | The approach allows for easy, correct and timely data collection with minimal missingness and manual labour. |


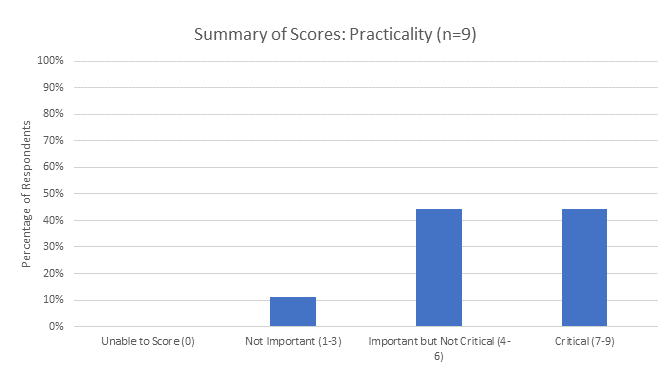


Comments on scoring:

| **Comment** | **Score Given** |
| --- | --- |
| More generally, ANY outcome that poses undue burden on respondents/study subjects is problematic. Other outcomes that involve a huge amount of work to collect can also become infeasible. | 8 |

Mechanistic Plausibility

| The approach enables a reasonable causal interpretation regarding the effect of an intervention. |
| --- |

Proposed attributes from Round 1 contributing to our definition:

| **Proposed Attribute Name** | **Definition** |
| --- | --- |
| Causality | A true causal interpretation must be possible - for example, excluding a large proportion of participants due to e.g. death, when this may be affected by the intervention, leads to results that are not fully interpretable from a causal point of view |
| Mechanistic Relevance | The outcome reflects treatment responsiveness of the underlying mechanism targeted by treatment |
| Plausibility | The outcome could reasonably be explained by the intervention. |


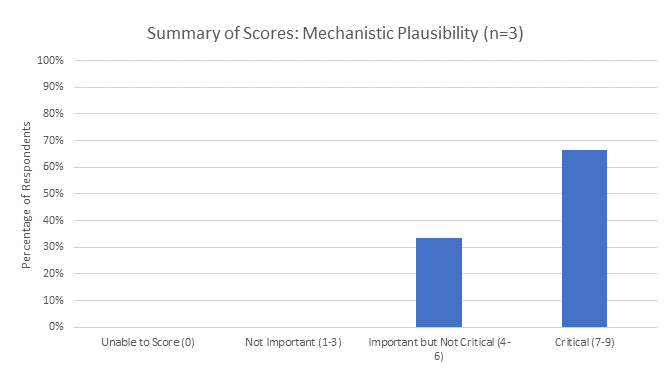


Comments on scoring:

| **Comment** | **Score Given** |
| --- | --- |
| Guards against Type 1 errors out of the blue...or at the very least, provokes a discussion of new paradigms. | 8 |

Statistical Simplicity

| The approach relies on a limited number of assumptions to estimate the effect of an intervention. |
| --- |

Proposed attributes from Round 1 contributing to our definition:

| **Proposed Attribute Name** | **Definition** |
| --- | --- |
| Robustness | The approach makes limited assumptions - or is less sensitive to deviations from its assumption. |
| Usability/Applicability | How easy it is to use the statistical method with common software, and how widely it can be used in different situations. This looks at how straightforward it is to use, the clear instructions for doing the analysis, making sure the basic assumptions are met, and providing different methods when necessary. |


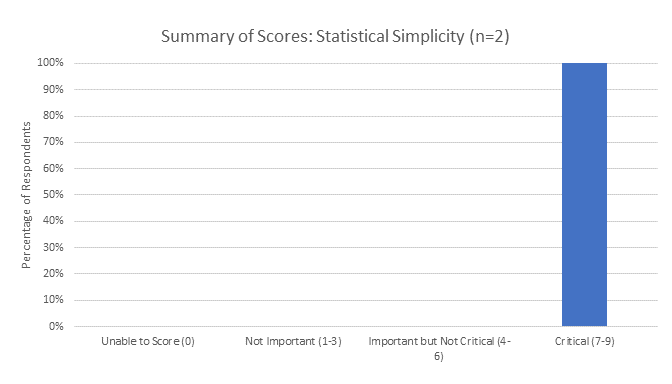


Comments on scoring:

| **Comment** | **Score Given** |
| --- | --- |
| Some statistical methods make strong unverifiable assumptions. For primary analysis want to limit strong assumptions. | 8 |

Attributes Dropped Following Round 1:

Based on your responses in Round 1, we have dropped Comparability and Familiarity from Round 2.

Comparability


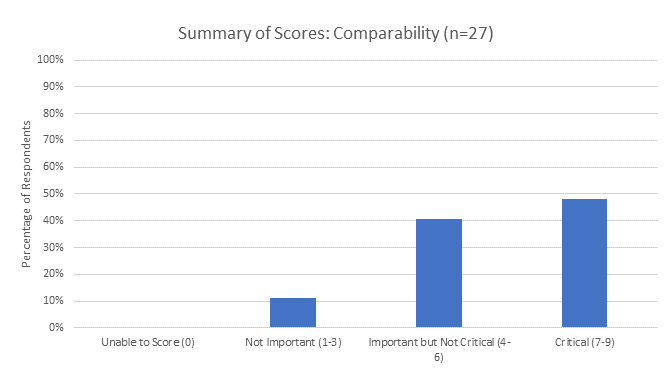


Familiarity


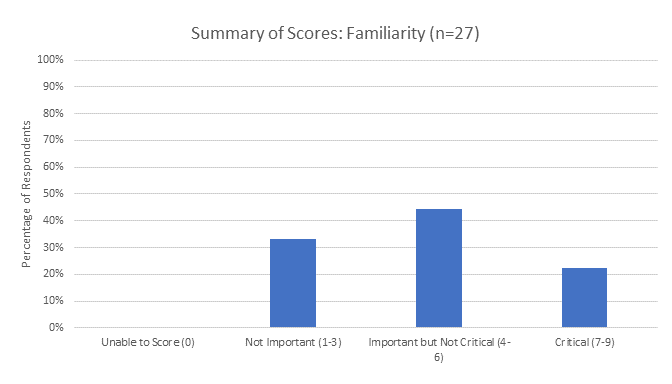


1. List of participants

Christine Adrion, PhD, MPH

Andrew D. Althouse, PhD

Derek C. Angus, MD, MPH

Paula Blonski

Elizabeth Colantuoni, PhD

Victoria Cornelius, PhD

Suzie Cro, PhD

Martha A.Q. Curley, PhD, RN, FAAN

Darren L Dahly, PhD

Eddy Fan, MD, PhD

Ewan Goligher MD, PhD

Anders Granholm, MD

Kimberley J. Haines, PhD, BHSc (Physio)

Anna Heath, PhD, MMath

Thomas Heymann, MBA

Carol L. Hodgson, PhD

Catherine L. Hough, MD MSc,

Leila Janani, PhD

Benjamin Skov Kaas-Hansen, MD, MSc, PhD

Rhonda E. Monroe, MBA

Vincent Mor, PhD

Tim P. Morris, PhD

Nicolas Paul, MD, MSc

Eileen Rubin, JD

Yael Schenker, MD, MAS

Norman Stockbridge, MD, PhD

B. Taylor Thompson, MD

Nadir Yehya, MD, MSCE

Fernando G Zampieri, MD, PhD

1. One participant responded to Round 1 after this Executive Summary was finalized but before Round 2 was opened. All responses received are included in the results reported in this article; the additional response did not change the overall distribution of scores or other results summarized in the Executive Summary. [↑](#footnote-ref-1)
